# Relationship between the weight-adjusted-waist index and urinary incontinence in women: A cross-sectional study of NHANES 2007 to 2020

**DOI:** 10.1097/MD.0000000000042996

**Published:** 2025-06-20

**Authors:** Weixing Jing, Cailiu Wei, Yiqi Huang, Tianxiao Fu, Weigang Shen, Weicheng Xiao

**Affiliations:** a Department of Emergency, Shaoxing Second Hospital, Shaoxing, Zhejiang, China; b Affiliated Hospital of Youjiang Medical University for Nationalities, Baise, Guangxi, China; c Department of Nephrology, Shaoxing Second Hospital, Shaoxing, Zhejiang, China; d Department of Traditional Chinese Medicine, The First Affiliated Hospital of Zhejiang University, Hangzhou, Zhejiang, China; e Department of Blood Purifying Center, Shaoxing Second Hospital, Shaoxing, Zhejiang, China.

**Keywords:** cross-sectional study, NHANES, obesity, urinary incontinence (UI), weight-adjusted-waist index (WWI)

## Abstract

This study investigated the relationship between the weight-adjusted-waist index (WWI), calculated as waist circumference (WC) divided by the square root of weight, and the risk of 3 types of urinary incontinence (UI) in women. Data from the National Health and Nutrition Examination Survey spanning 2007 to 2020 were analyzed, including 7236 female participants aged 20 years and older. UI types (stress UI [SUI], urgency UI [UUI], and mixed UI [MUI]) were classified based on self-reported questionnaires. Multivariate logistic regression models were used to assess the association between WWI and UI, adjusting for covariates such as age, race, marital status, education level, and comorbidities. Subgroup analyses were conducted to evaluate the consistency of associations across different age groups, body mass index (BMI) categories, and racial backgrounds. Additionally, dose–response relationships were examined, and receiver operating characteristic curve analysis was performed to compare the predictive ability of WWI, BMI, and WC for UI. The results revealed that the prevalence rates of SUI, UUI, and MUI were 47.55%, 29.09%, and 18.14%, respectively. Higher WWI was significantly associated with increased risks of all 3 UI types. In the fully adjusted model (Model 4), the odds ratios for SUI, UUI, and MUI were 1.28 (95% confidence interval [CI]: 1.18–1.37), 1.17 (95% CI: 1.08–1.28), and 1.23 (95% CI: 1.13–1.34), respectively. Subgroup analyses confirmed consistent associations across various demographic and clinical subgroups. Receiver operating characteristic curve analysis demonstrated that WWI had superior discrimination ability compared to BMI and WC, with higher area under the curve (AUC) values for SUI (AUC = 0.601), UUI (AUC = 0.630), and MUI (AUC = 0.628). In conclusion, this study highlights a significant association between higher WWI and increased risks of SUI, UUI, and MUI in women. WWI may serve as a more effective anthropometric indicator for assessing UI risk compared to traditional measures like BMI and WC, offering potential utility in clinical and public health settings for identifying individuals at higher risk of UI and guiding targeted prevention strategies.

## 1. Introduction

Urinary incontinence (UI), a multifaceted condition is defined as involuntary loss of urine, presents a significant health challenge affecting a wide spectrum of individuals, irrespective of gender and age.^[1,2]^ The frequency of UI in older adult women ranges from 25% to 45% overall, with institutionalized older women having a greater prevalence than community-dwelling older women. Quality of life suffers as a consequence of UI, with greater impact as the severity of leakage increases.^[1,3]^ It can lead to psychological stress, and social isolation, and significantly impact an individual’s quality of life. The typology of UI is dissected into 3 principal categories, each distinguished by unique etiological factors and pathophysiological mechanisms: stress UI (SUI), urgency UI (UUI), and mixed UI (MUI).^[4]^

Body mass index (BMI), a ubiquitous but sometimes contentious gauge of obesity, derived from a mathematical formula where one’s mass in kilograms is divided by their height in square meters squared (kg/m^2^), has sparked debates regarding its efficacy as a barometer of metabolic health.^[5]^ This skepticism stems from BMI’s inherent limitations, notably its inability to discriminate between muscular and adipose tissues and to differentiate central from peripheral adiposity.^[6,7]^ Nevertheless, the role of obesity as an overarching risk factor for UI, particularly in women, stands undisputed.^[1,8]^ BMI, as an indicator of obesity, has been shown in studies to be significantly associated with an increased risk of urinary incontinence as it rises.^[8,9]^ Women who increase their BMI by 5 units will likely experience 20% to 70% more stress incontinence.^[10]^ In an endeavor to transcend the constraints of BMI, a novel metric, the “weight-adjusted-waist index (WWI),” has emerged. This innovative index, recalibrating waist circumference (WC) in the context of body weight, is postulated to offer a more nuanced and precise reflection of the fat-to-muscle mass ratio, with a specific focus on central obesity.^[11]^

Nowadays, numerous studies have investigated the clinical factors associated with the occurrence and severity of UI in women. However, there are no relevant studies to verify the results of WWI. This study was designed to determine the relationship between WWI and UI in a US female population.

## 2. Methods

### 2.1. Study population

**Figure 1.**
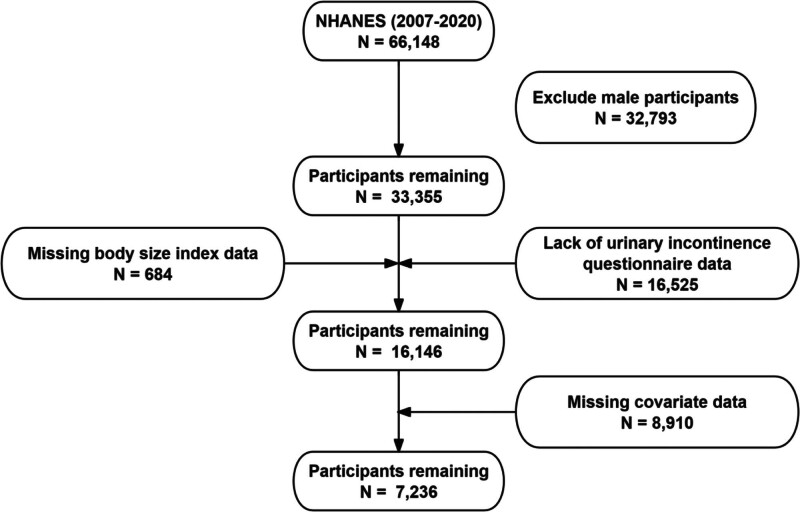
The selection process of NHANES 2007 to 2020. NHANES = National Health and Nutrition Examination Survey.

The National Health and Nutrition Examination Survey (NHANES) stands as a beacon of data richness and national representativeness, meticulously orchestrated by the Centers for Disease Control and Prevention in the United States. NHANES research programs are reviewed and approved by the National Health Statistics Research Council Center.^[12]^ NHANES collects data on people’s health, nutrition, and lifestyle through surveys, interviews, checkups, and lab tests. It covers topics like chronic illnesses, diet, physical activity, and environmental exposures. This dataset serves as a vital resource for monitoring public health trends, shaping health policies, and guiding clinical and community interventions. The inclusion and exclusion criteria for participants in this study are shown in Figure 1.

### 2.2. Assessment of WWI and UI

The present analysis produced the following results: MUI, SUI, and UUI. If they were asked, “During the past 12 months, have you leaked or lost control of even a small amount of urine with an activity like coughing, lifting, or exercising?” The individual is classified as SUI if their response to the question is yes. If they responded to the query “During the past 12 months, have you leaked or lost control of even a small amount of urine with an urge or pressure to urinate and you couldn’t get to the toilet fast enough?” The participant in this problem is defined as UUI. Participants were categorized as MUI if they provided a yes response to both questions. Previous studies have shown that the evaluation of UI types by self-reported questionnaires is considered valid and reliable.^[13]^ WWI is a weight-based anthropometric WC measure used to evaluate obesity, specifically central obesity. It was selected as it better represents abdominal fat distribution compared to other indices, as it combines both waist circumference and weight. A higher WWI score has been shown to correlate with a higher level of obesity. Weight and WC data were obtained at the Mobile Examination Center by trained health workers who followed standardized procedures for body measurements. In our study, WWI was used as an exposure variable. The WWI was calculated using the following formula:


$$\begin{array}{l} WWI\;\;\;=\;\;\;WC\;\;\;\left(cm\right)/\sqrt{Kg} \end{array}$$

### 2.3. Other clinical covariates

The selection of covariates was based on previously published literature.^[14,15]^ We included age, race (White, Black, and Other), marital status (married/living with a partner, single/divorced/widowed), educational level (less than high school, high school or equivalent, college or above), smoking status (never, former, and current), drinking status (never, former, and current), hypertension (no, yes), diabetes (no, yes), number of vaginal deliveries (0, 1–2, 3–4, and ≥5), urinary creatinine, and the poverty–income ratio (PIR), a measure of socioeconomic status, calculated as the ratio of household income to the poverty threshold, and categorized as ≤1.30, 1.31 to 3.50, and >3.50.

### 2.4. Statistical analysis

The accuracy of the data was increased by using suitable sample weights for weighted analysis in accordance with NHANES rules, which lessened the influence of the complicated multi-stage sampling design utilized by the NHANES. The weighted mean ± standard error for continuous variables and the weighted percentage (%) for categorical variables are used to express demographic features. A weighted Chi-square test and a weighted *T* test were used to assess the study population’s baseline characteristics. WWI and SUI, UUI, and MUI were compared using weighted multivariate and weighted univariate logistic regression models. Four weighted logistic regression models were built: there was no variable adjustment for Model 1, age, race, and marital status. Models 2 and 3 included adjustments for age, race, education level, recreational activities, smoking and drinking status, and number of vaginal deliveries. Diabetes, hypertension, and urine creatinine are further adjusted for in Model 4. Furthermore, we showed dose-response associations between WWI and the risk of SUI, UUI, and MUI in Model 4 using a weighted restricted cubic spline (RCS) plot (with 3 knots). RCS flexibly captures nonlinear associations, avoiding excessive assumptions about data patterns, making the results more biologically meaningful and robust. After stratifying according to age, race, and BMI, then looked for differences in the associations between the subgroups using interaction analyses. Receiver operating characteristics (ROC) curves were used to assess the ability of different anthropometric indicators to identify individual incontinence. R software was used to sort and analyze all statistical analyses in this study (R4.2.3). *P* < .05 on both sides was regarded as statistically significant.

## 3. Result

### 3.1. Characteristics of participants

Included in the study were 7236 female participants who were 20 years of age or older, with a mean weighted age of 49.23 ± 0.29 years. The prevalence rates of SUI, UUI, and MUI were 47.55%, 29.09%, and 18.14%, respectively. The participant characteristics are categorized by WWI quartiles in Table 1. The first, second, third, and fourth quartiles’ WWI ranges were 8.79 to 10.72, 10.73 to 11.27, 11.28 to 11.84, and 11.85 to 15.39, respectively. Participants in the highest WWI quartile were more likely to be single/divorced/widowed, hypertensive, physically inactive, less educated, and have a lower socioeconomic status (PIR ≤ 1.30). Additionally, individuals in the highest WWI quartile had greater prevalence rates of SUI (56.04%), UUI (39.40%), and MUI (25.76%) compared to those in the other quartiles.

**Table 1:** Demographic characteristics stratified by quartile of WWI (N = 7236).

Characteristic	Total	Quartile 1 8.79–10.72	Quartile 2 10.73–11.27	Quartile 3 11.28–11.84	Quartile 4 11.85–15.39	*P*-value
Age	49.23 (0.29)	42.75 (0.40)	48.43 (0.39)	51.38 (0.43)	56.26 (0.54)	**<.0001**
Creatinine urine(mg/dl)	105.45 (1.43)	104.59 (2.37)	104.43 (2.19)	106.18 (2.43)	107.00 (2.56)	.83
Race						**<.0001**
White	2748 (65.05)	734 (66.75)	682 (64.88)	619 (61.96)	713 (66.36)	
Black	1811 (13.36)	546 (15.28)	445 (12.57)	445 (13.86)	375 (11.33)	
Mexican	1043 (7.92)	142 (4.78)	237 (7.57)	323 (10.06)	341 (10.10)	
Other	1634 (13.67)	387 (13.19)	447 (14.97)	420 (14.13)	380 (12.20)	
Marital status						**<.0001**
Married/living with partner	4144 (64.60)	1063 (65.52)	1094 (68.89)	1059 (64.75)	928 (58.08)	
Single/divorced/widowed	3092 (35.40)	746 (34.48)	717 (31.11)	748 (35.25)	881 (41.92)	
Education level						**<.0001**
Less than high school	1518 (13.26)	238 (8.63)	333 (11.54)	398 (14.80)	549 (19.66)	
High school or equivalent	1557 (21.97)	340 (17.69)	370 (20.50)	415 (22.81)	432 (28.37)	
College or above	4161 (64.77)	1231 (73.67)	1108 (67.96)	994 (62.39)	828 (51.96)	
PIR						**<.0001**
≤1.30	2489 (22.78)	529 (19.17)	578 (20.54)	637 (24.02)	745 (28.81)	
1.31–3.50	2625 (34.59)	595 (29.87)	646 (32.34)	657 (34.94)	727 (43.02)	
>3.50	2122 (42.63)	685 (50.96)	587 (47.13)	513 (41.04)	337 (28.17)	
Recreational activity						**<.0001**
No activity	3989 (48.11)	811 (35.96)	926 (45.86)	1052 (51.46)	1200 (62.90)	
Moderate	2001 (30.61)	454 (28.97)	563 (32.64)	520 (33.70)	464 (26.97)	
Vigorous	1246 (21.29)	544 (35.07)	322 (21.50)	235 (14.84)	145 (10.13)	
Smoking status						**.04**
Never	4355 (57.56)	1123 (60.35)	1107 (58.10)	1089 (56.73)	1036 (54.20)	
Former	1446 (23.02)	285 (19.55)	354 (23.16)	393 (24.60)	414 (25.62)	
Now	1435 (19.42)	401 (20.09)	350 (18.73)	325 (18.67)	359 (20.19)	
Drinking status						**<.0001**
No	1218 (11.88)	204 (9.09)	259 (9.83)	344 (12.14)	411 (17.65)	
Former	867 (9.83)	153 (6.95)	195 (8.52)	220 (11.07)	299 (13.78)	
Now	5151 (78.30)	1452 (83.95)	1357 (81.65)	1243 (76.78)	1099 (68.57)	
Hypertension						**<.0001**
No	4182 (62.62)	1408 (80.76)	1159 (68.58)	920 (55.06)	695 (40.11)	
Yes	3054 (37.38)	401 (19.24)	652 (31.42)	887 (44.94)	1114 (59.89)	
Diabetes						**<.0001**
No	5910 (85.68)	1726 (96.73)	1613 (91.33)	1412 (82.31)	1159 (68.23)	
Yes	1326 (14.32)	83 (3.27)	198 (8.67)	395 (17.69)	650 (31.77)	
Number of deliveries						**<.0001**
0	577 (9.01)	242 (14.19)	136 (7.52)	120 (7.86)	79 (5.38)	
1–2	3580 (54.71)	949 (54.75)	947 (59.16)	895 (54.45)	789 (49.55)	
3–4	2410 (30.22)	523 (26.97)	597 (28.62)	613 (31.22)	677 (35.26)	
≥5	669 (6.06)	95 (4.09)	131 (4.70)	179 (6.47)	264 (9.81)	
SUI						**<.0001**
No	3994 (52.45)	1180 (62.67)	1018 (50.40)	954 (50.38)	842 (43.96)	
Yes	3242 (47.55)	629 (37.33)	793 (49.60)	853 (49.62)	967 (56.04)	
UUI						**<.0001**
No	4957 (70.91)	1406 (79.37)	1313 (75.17)	1172 (65.53)	1066 (60.60)	
Yes	2279 (29.09)	403 (20.63)	498 (24.83)	635 (34.47)	743 (39.40)	
MUI						**<.0001**
No	5846 (81.86)	1601 (89.00)	1498 (83.53)	1414 (78.47)	1333 (74.24)	
Yes	1390 (18.14)	208 (11.00)	313 (16.47)	393 (21.53)	476 (25.76)	

Bold values indicates significant difference between the two groups.

MUI = mixed urinary incontinence, PIR = poverty–income ratio, SUI = stress urinary incontinence, UUI = urgency urinary incontinence, WWI = weight-adjusted-waist index.

### 3.2. The relationship between WWI and UI

**Figure 2.**
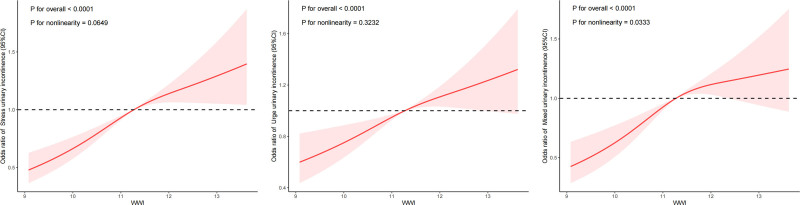
Dose–response relationship analysis between WWI and urinary incontinence. Restricted cubic spline (RCS) regression with 3 knots (10th, 50th, and 90th percentiles) to examine the nonlinearity of the association between WWI and UI. RCS regression was adjusted for age, race, marital status, education level, PIR, recreational activity, smoking status, drinking status, number of vaginal deliveries, hypertension, diabetes, and creatinine urine (Model 4). The red solid line represents ORs, red shaded region represents 95 % CI. CI = confidence interval, MUI = mixed urinary incontinence, OR = odds ratio, SUI = stress urinary incontinence, UUI = urgency urinary incontinence, WWI = weight-adjusted-waist index.

A higher risk of SUI, UUI, and MUI was linked to a higher WWI, as demonstrated by weighted multivariate logistic regression analysis (Table 2). In Model 1, there was a significant correlation (*P* < .0001) between the variables, with odds ratios (OR) of 1.38 (95% confidence interval [CI] = 1.30–1.47) for SUI, 1.60 (95% CI = 1.50–1.71) for UUI, and 1.60 (95% CI = 1.50–1.71) for MUI. Furthermore, even after accounting for all confounders, the results from Model 4 remained statistically significant (SUI: OR = 1.28, 95% CI = 1.18–1.37; UUI: OR = 1.17, 95% CI = 1.08–1.28; MUI: OR = 1.23, 95% CI = 1.13–1.34, both *P* < .0001). To facilitate future analysis, WWI was transformed from a continuous variable to a categorical variable (quartile). In Model 4, we found that the Q4 group of WWI had 1.71-fold, 1.43-fold, and 1.65-fold higher risk of SUI, UUI, and MUI, respectively, compared to the Q1 group. Figure 2 illustrates the dose–response relationship between WWI and different types of UI, including SUI, UUI, and MUI. Across all 3 types, a higher WWI is associated with an increased risk of UI, with significant overall associations (*P* < .0001). While the relationships for SUI and UUI appear linear, MUI shows some evidence of nonlinearity (*P* = .0333). The findings suggest that greater central adiposity, as reflected by WWI, may elevate the risk of UI, after adjusting for all potential confounders.

**Table 2:** Logistic regression analysis of WWI and urinary incontinence.

	SUI		UUI		MUI	
	OR (95%CI)	*P*-value	OR (95%CI)	*P*-value	OR (95%CI)	*P*-value
*Model 1*						
WWI	1.42 (1.31, 1.54)	<.0001	1.54 (1.42, 1.67)	<.0001	1.58 (1.45, 1.73)	<.0001
*Stratified by WWI quartiles*						
Quartile 1	1		1		1	
Quartile 2	1.65 (1.37, 1.99)	<.0001	1.27 (1.05, 1.53)	.01	1.59 (1.27, 2.01)	<.001
Quartile 3	1.65 (1.39, 1.97)	<.0001	2.02 (1.64, 2.50)	<.0001	2.22 (1.74, 2.84)	<.0001
Quartile 4	2.14 (1.76, 2.60)	<.0001	2.50 (2.08, 3.01)	<.0001	2.81 (2.25, 3.51)	<.0001
*Model 2*						
WWI	1.32 (1.20, 1.45)	<.0001	1.23 (1.12, 1.34)	<.0001	1.33 (1.21, 1.47)	<.0001
*Stratified by WWI quartiles*						
Quartile 1	1		1		1	
Quartile 2	1.55 (1.27, 1.88)	<.0001	1.03 (0.83, 1.26)	.8	1.36 (1.07, 1.74)	.01
Quartile 3	1.51 (1.25, 1.82)	<.0001	1.48 (1.17, 1.87)	.001	1.75 (1.36, 2.26)	<.0001
Quartile 4	1.81 (1.45, 2.26)	<.0001	1.54 (1.27, 1.87)	<.0001	1.94 (1.52, 2.47)	<.0001
*Model 3*						
WWI	1.31 (1.19, 1.44)	<.0001	1.17 (1.06, 1.29)	.002	1.26 (1.13, 1.40)	<.0001
*Stratified by WWI quartiles*						
Quartile 1	1		1		1	
Quartile 2	1.48 (1.21, 1.82)	<.001	1.03 (0.83, 1.28)	.78	1.32 (1.04, 1.68)	.03
Quartile 3	1.50 (1.25, 1.79)	<.0001	1.50 (1.17, 1.91)	.002	1.71 (1.31, 2.23)	<.001
Quartile 4	1.86 (1.49, 2.32)	<.0001	1.55 (1.24, 1.93)	<.001	1.84 (1.41, 2.40)	<.0001
*Model 4*						
WWI	1.27 (1.16, 1.40)	<.0001	1.19 (1.07, 1.32)	<.0001	1.24 (1.11, 1.39)	<.001
*Stratified by WWI quartiles*						
Quartile 1	1		1		1	
Quartile 2	1.40 (1.17, 1.67)	<.001	1.02 (0.82, 1.26)	.86	1.30 (1.02, 1.65)	.03
Quartile 3	1.45 (1.19, 1.77)	<.001	1.45 (1.13, 1.86)	.004	1.62 (1.25, 2.10)	<.001
Quartile 4	1.65 (1.32, 2.08)	<.0001	1.45 (1.15, 1.83)	.002	1.67 (1.28, 2.17)	<.001

Model 1: unadjusted.

Model 2: adjusted for age and race.

Model 3: adjusted for age, race, marital status, education level, PIR, recreational activity, smoking status, drinking status, and number of deliveries.

Model 4: further adjusted for hypertension, diabetes, and creatinine urine.

Covariates were selected based on prior literature and their potential associations with both WWI and urinary incontinence.

Model 4 was considered the primary model, providing the most comprehensively adjusted estimates.

Odds ratios (ORs) reflect the relative odds of urinary incontinence per unit increase in WWI.

MUI = mixed urinary incontinence, SUI = stress urinary incontinence, UUI = urgency urinary incontinence, WWI = weight-adjusted-waist index.

### 3.3. Subgroup analysis

Subgroup analyses stratified according to age (20–39 years, 40–59 years, ≥60 years), BMI (< 30, ≥30), and race (White, Black, Mexican, and other race) were performed (Table 3). After adjusting for covariates, we found no significant differences in WWI and UI within these subgroups (all *P* for interaction >.05). Specifically, the association between WWI and UI risk was consistent across subgroups of age, BMI, and race.

**Table 3:** Subgroups analysis stratified by age, BMI, and race.

WWI	SUI		UUI		MUI	
	OR (95% CI)	*P* for interaction	OR (95% CI)	*P* for interaction	OR (95% CI)	*P* for interaction
Age		.31		.41		.75
20–39	1.41 (1.21, 1.65)		1.19 (1.02, 1.38)		1.22 (1.01, 1.47)	
40–59	1.19 (1.03, 1.38)		1.14 (0.98, 1.33)		1.26 (1.04, 1.53)	
≥ 60	1.26 (1.04, 1.52)		1.27 (1.04, 1.56)		1.27 (1.04, 1.56)	
BMI		.15		.81		.29
< 30	1.30 (1.14, 1.49)		1.16 (0.97, 1.38)		1.28 (1.02, 1.59)	
≥ 30	1.06 (0.90, 1.24)		1.06 (0.92, 1.22)		1.06 (0.90, 1.25)	
Race		.39		.78		.25
White	1.22 (1.08, 1.39)		1.15 (0.99, 1.34)		1.18 (1.01, 1.39)	
Black	1.30 (1.09, 1.54)		1.14 (1.02, 1.28)		1.33 (1.11, 1.60)	
Mexican	1.14 (0.91, 1.43)		1.09 (0.88, 1.36)		0.96 (0.75, 1.25)	
Other	1.58 (1.29, 1.94)		1.48 (1.18, 1.86)		1.71 (1.30, 2.23)	

Analyses were adjusted for age, race, marital status, education level, PIR, recreational activity, smoking status, drinking status, number of vaginal deliveries, hypertension, diabetes, and creatinine urine.

BMI = body mass index, MUI = mixed urinary incontinence, SUI = stress urinary incontinence, UUI = urgency urinary incontinence, WWI = weight-adjusted-waist index.

### 3.4. Discrimination ability of different anthropometric

**Figure 3.**
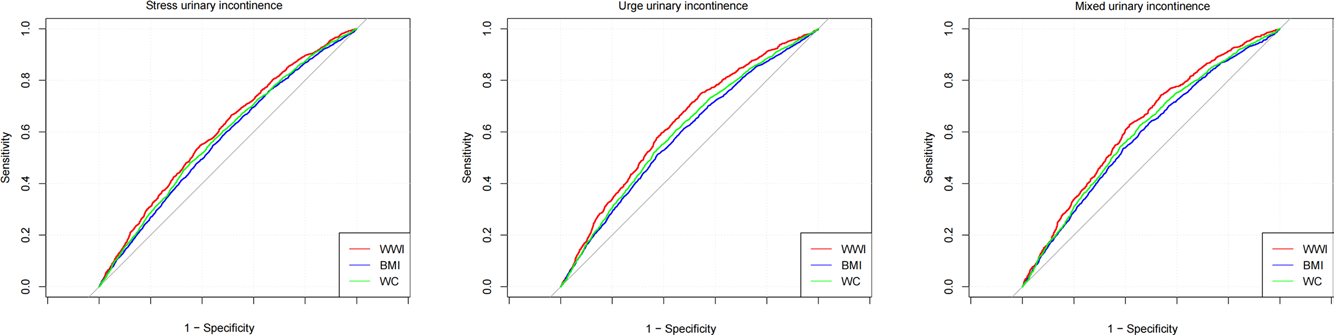
ROC curves of anthropometric indices for discriminating urinary incontinence. AUC = area under the curve, BMI = body mass index, ROC = receiver operating characteristic, WC = waist circumference, WWI = weight-adjusted-waist index.

The discrimination abilities of the WWI, BMI, and WC in predicting urinary incontinence types (SUI, UUI, and MUI) were assessed using ROC curve analysis. As shown in Figure 3 and Table 4, the area under the curve (AUC) values for WWI, BMI, and WC in predicting SUI, UUI, and MUI were compared.

**Table 4:** The adiposity indicators for predicting urinary incontinence.

Test	AUC (95% CI)	Cutoff value	Specificity	Sensitivity	*P* for difference in AUC
Stress urinary incontinence					
** **WWI	0.601 (0.584, 0.618)	11.412	0.548	0.608	Reference
** **BMI	0.569 (0.556, 0.582)	28.795	0.585	0.521	.004
** **WC	0.581 (0.568, 0.594)	101.55	0.480	0.646	.023
Urge urinary incontinence					
** **WWI	0.630 (0.612, 0.648)	11.086	0.748	0.457	Reference
** **BMI	0.585 (0.571, 0.600)	30.785	0.513	0.625	<.0001
** **WC	0.600 (0.586, 0.614)	97.850	0.606	0.554	.001
Mixed urinary incontinence					
** **WWI	0.628 (0.607, 0.649)	11.412	0.629	0.584	Reference
** **BMI	0.589 (0.572, 0.605)	30.568	0.536	0.609	.004
** **WC	0.603 (0.587, 0.619)	98.150	0.626	0.543	.035

AUC = area under the curve, BMI = body mass index, ROC = receiver operating characteristic, WC = waist circumference, WWI = weight-adjusted-waist index.

For SUI, WWI had an AUC of 0.601 (95% CI: 0.584–0.618), significantly outperforming BMI (AUC 0.569, 95% CI: 0.556–0.582, *P* = .004) and WC (AUC 0.581, 95% CI: 0.568–0.594, *P* = .023). In predicting UUI, WWI also showed the highest AUC of 0.630 (95% CI: 0.612–0.648), with BMI and WC showing lower AUCs of 0.585 (95% CI: 0.571–0.600) and 0.600 (95% CI: 0.586–0.614), respectively (*P* < .0001 for BMI vs WWI and *P* = .001 for WC vs WWI). Similarly, for MUI, WWI demonstrated the best discrimination ability, with an AUC of 0.628 (95% CI: 0.607–0.649), while BMI and WC had AUCs of 0.589 (95% CI: 0.572–0.605) and 0.603 (95% CI: 0.587–0.619), respectively (*P* = .004 for BMI vs WWI and *P* = .035 for WC vs WWI).

## 4. Discussion

In this study, we examined the relationships between 3 categories of UI and WWI in the female population using the 2007 to 2020 NHANES datasets. Our study found a significant link between WWI and UI. We used both univariate and multivariate logistic regression analyses and found that WWI was associated with an elevated risk of SUI, UUI, and MUI. Our dose–response analyses established a positive linear correlation between WWI and UI. Our subgroup analysis indicated that the relationship was stable across different subgroups.

This study and other studies have confirmed a high correlation between obesity and the incidence of UI. Estimates of prevalence for middle-aged women range from 30% to 40%, and for elderly women, they rise to almost 50%.^[16]^ After a thorough analysis, there is compelling evidence to show that abdominal obesity and the waist–hip ratio, in addition to BMI, may independently raise the risk of incontinence in women.^[17]^ Odds ratios between overweight and obese women and those with normal weight were found to be higher in the cross-sectional surveys. Based on data from the EPINCONT study, a 30% BMI reduction for all of these obese women would result in a considerable decrease in the percentage of incontinent women.^[18]^ A research of 136 women with an average BMI of 28 kg/m^2^ found that when BMI increased, so did intra-abdominal pressure and intravesical pressure.^[19,20]^ In the PRIDE research, for instance, 338 obese women who were overweight and had UI at least 10 times a week were randomly assigned to receive either a 6-month weight reduction intervention or a controlled education program.^[21]^ Over the course of 6 months, the intervention group shed 7.8 kg whereas the control group shed only 1.5 kg. Due to this disparity, the intervention group’s weekly incontinence decreased by 47% while the control group’s decreased by 28%.

The incidence of UI 20 years after a single delivery was examined by Gyhagen et al.^[22,23]^ Another important risk factor in the association between the prevalence of UI and pregnancy and delivery is maternal weight. A multivariate model analysis indicates that the likelihood of UI increases by 8% for every additional BMI unit. The rates of UI in both distribution types are impacted by this phenomenon.

Our subgroup analysis showed that the association between WWI and UI remained stable across races and age groups. These results suggest that WWI may affect UI through some relatively common biological mechanisms, and these mechanisms have certain commonalities in different populations. Obesity increases intra-abdominal pressure, which weakens the pelvic muscles and pelvic innervation, according to research by Ramalingam et al.^[24]^ This pressure exacerbates detrusor instability and causes SUI by increasing pressure in the bladder and urethral mobility.^[25]^ Obese people have dysregulated levels of inflammatory cytokines and factors, including interleukin 6 and tumor necrosis factor-alpha. It has been hypothesized that through altering collagen metabolism, the oxidative stress caused by adipose tissue will raise the frequency and severity of incontinence.^[26]^ Obesity also leads to mechanical stress on the pelvic floor, further weakening the pelvic muscles and impairing neuromuscular coordination. The increased abdominal fat places pressure on the pelvic organs, leading to bladder and urethral dysfunction, which contributes to both SUI and urgency-related incontinence. Furthermore, chronic low-grade inflammation in obesity, driven by pro-inflammatory cytokines, disrupts the extracellular matrix, including collagen, which is essential for maintaining the strength and integrity of the pelvic floor. These factors create a complex mechanism that predisposes individuals with obesity to urinary incontinence.

In the context of incontinence, waist circumference: which is indicated by a high waist–hip ratio accurately assesses fat distribution; BMI does not. Data from randomized trials indicated that while the waist–hip ratio was not proven to be a risk factor for stress incontinence, it was an independent risk factor for UUI or MUI.^[27]^ In Nurses’ Health Study, higher BMI and waist circumference increase the risk of incontinence.^[28]^ While waist circumference did not correlate with BMI, it did correlate with UUI and MUI. Although other common indices, such as waist–hip ratio and BMI, are often used to assess central obesity, WWI (a relatively novel index combining weight and waist circumference) offers a potential advantage by reflecting both muscle mass and fat mass in relation to central obesity. However, it should be noted that while WHR and other indices have been widely used in clinical research, the application of WWI in intervention studies remains limited. In our analysis, ROC curve showed that WWI had obvious advantages over traditional obesity indexes such as BMI and WC in predicting urinary incontinence. Further studies are needed to evaluate the clinical utility of WWI in larger intervention trials.

There are various drawbacks to this study. First, the cross-sectional nature of the current study precludes the inference of causality. While our study demonstrates a significant association between WWI and UI, we cannot establish a direct cause-and-effect relationship. Future longitudinal studies are needed to validate these findings and explore the temporal relationship between WWI and UI. Such studies would help determine whether WWI serves as an early predictor for UI and provide more robust evidence for preventive interventions. Second, when survey participants self-report a history of UI, a smaller sample size than necessary may result. Third, unlike BMI and WC, WWI is a relatively novel measure of obesity and has not been extensively utilized to evaluate obesity or central obesity in normal or clinical contexts. Lastly, although we adjusted for several potential confounders, some residual confounders may still influence the observed associations.

## 5. Conclusion

This study highlights a significant association between higher WWI and increased risks of SUI, UUI, and MUI in women. WWI may serve as a more effective anthropometric indicator for assessing UI risk compared to traditional measures like BMI and WC, offering potential utility in clinical and public health settings for identifying individuals at higher risk of urinary incontinence and guiding targeted prevention strategies.

## Author contributions

**Conceptualization:** Weixing Jing, Yiqi Huang, Weicheng Xiao.

**Data curation:** Weixing Jing, Yiqi Huang, Tianxiao Fu, Weigang Shen, Weicheng Xiao.

**Formal analysis:** Weixing Jing, Yiqi Huang, Tianxiao Fu, Weigang Shen, Weicheng Xiao.

**Methodology:** Tianxiao Fu, Weigang Shen.

**Software:** Cailiu Wei, Weigang Shen.

**Supervision:** Cailiu Wei.

**Validation:** Cailiu Wei.

**Writing – original draft:** Weixing Jing, Yiqi Huang, Weicheng Xiao.

**Writing – review & editing:** Weixing Jing, Cailiu Wei, Yiqi Huang, Weicheng Xiao.

## References

[1] Coyne KS Kvasz M Ireland AM Milsom I Kopp ZS Chapple CR. Urinary incontinence and its relationship to mental health and health-related quality of life in men and women in Sweden, the United Kingdom, and the United States. Eur Urol. 2012;61:88–95. 21831517 10.1016/j.eururo.2011.07.049

[2] Omli R Hunskaar S Mykletun A Romild U Kuhry E. urinary incontinence and risk of functional decline in older women: data from the Norwegian HUNT-Study. BMC Geriatr. 2013;13:47. 23678851 10.1186/1471-2318-13-47 PMC3660293

[3] Irwin DE Kopp ZS Agatep B Milsom I Abrams P. Worldwide prevalence estimates of lower urinary tract symptoms, overactive bladder, urinary incontinence and bladder outlet obstruction: WORLDWIDE PREVALENCE OF LUTS. BJU International. 2011;108:1132–8. 21231991 10.1111/j.1464-410X.2010.09993.x

[4] Zhang D Gao L Jia Y. Construction of progress prediction model of urinary incontinence in elderly women: protocol for a multi-center, prospective cohort study. Int J Environ Res Public Health. 2022;19:734. 35055556 10.3390/ijerph19020734 PMC8775636

[5] Piché M-E Tchernof A Després J-P. obesity phenotypes, diabetes, and cardiovascular diseases. Circ Res. 2020;126:1477–500. 32437302 10.1161/CIRCRESAHA.120.316101

[6] Oliveros E Somers VK Sochor O Goel K Lopez-Jimenez F. The concept of normal weight obesity. Prog Cardiovasc Dis. 2014;56:426–33. 24438734 10.1016/j.pcad.2013.10.003

[7] Hainer V Aldhoon-Hainerová I. Obesity paradox does exist. Diabetes Care. 2013;36(Supplement_2):S276–81. 23882059 10.2337/dcS13-2023 PMC3920805

[8] Phelan S Kanaya AM Subak LL. Prevalence and risk factors for urinary incontinence in overweight and obese diabetic women. Diabetes Care. 2009;32:1391–7. 19487639 10.2337/dc09-0516 PMC2713631

[9] Moreno-Vecino B Arija-Blázquez A Pedrero-Chamizo R. Associations between obesity, physical fitness, and urinary incontinence in non-institutionalized postmenopausal women: the elderly EXERNET multi-center study. Maturitas. 2015;82:208–14. 26261038 10.1016/j.maturitas.2015.07.008

[10] Saei Ghare Naz M Ramezani Tehrani F Behroozi-Lak T Mohammadzadeh F Kholosi Badr F Ozgoli G. Polycystic ovary syndrome and pelvic floor dysfunction: a narrative review. Res Reports Urol. 2020;12:179–85. 10.2147/RRU.S249611 PMC7213900 32440514

[11] Park Y Kim NH Kwon TY Kim SG. A novel adiposity index as an integrated predictor of cardiometabolic disease morbidity and mortality. Sci Rep. 2018;8:16753. 30425288 10.1038/s41598-018-35073-4 PMC6233180

[12] Ahluwalia N Dwyer J Terry A Moshfegh A Johnson C. Update on NHANES dietary data: focus on collection, release, analytical considerations, and uses to inform public policy. Adv Nutr (Bethesda, Md.). 2016;7:121–34. 10.3945/an.115.009258 PMC4717880 26773020

[13] Weinberg AE Leppert JT Elliott CS. Biochemical measures of diabetes are not independent predictors of urinary incontinence in women. J Urol. 2015;194:1668–74. 26087382 10.1016/j.juro.2015.06.074

[14] Chen F Lin H Zhang Y Zhang Y Chu M Pan L. Impact of weight loss on the risk of urinary incontinence: the role of sex and body type. World J Urol. 2024;42:616. 39487931 10.1007/s00345-024-05333-2

[15] Li X Zhou W Hu G. The association between non-alcoholic fatty liver disease and urinary incontinence among adult females in the United States. BMC Public Health. 1373;24:1371. 10.1186/s12889-024-18578-8 PMC11110403 38778285

[16] Hunskaar S. A Systematic review of overweight and obesity as risk factors and targets for clinical intervention for urinary incontinence in women. Neurourol Urodyn. 2008;27:749–57. 18951445 10.1002/nau.20635

[17] Hunskaar S. A systematic review of overweight and obesity as risk factors and targets for clinical intervention for urinary incontinence in women. Neurourol Urodyn. 2008;27:749–57. 18951445 10.1002/nau.20635

[18] Ebbesen MH Hunskaar S Rortveit G Hannestad YS. Prevalence, incidence and remission of urinary incontinence in women: longitudinal data from the Norwegian HUNT Study (EPINCONT). BMC Urol. 2013;13:27. 23721491 10.1186/1471-2490-13-27 PMC3674916

[19] Subak LL Whitcomb E Shen H Saxton J Vittinghoff E Brown JS. Weight loss: a novel and effective treatment for urinary incontinence. J Urol. 2005;174:190–5. 15947625 10.1097/01.ju.0000162056.30326.83 PMC1557356

[20] Bump RC Sugerman HJ Fantl JA McClish DK. Obesity and lower urinary tract function in women: effect of surgically induced weight loss. Am J Obstet Gynecol. 1992;167:392–7; discussion 397. 1497041 10.1016/s0002-9378(11)91418-5

[21] Subak LL Wing R West DS. Weight loss to treat urinary incontinence in overweight and obese women. N Engl J Med. 2009;360:481–90. 19179316 10.1056/NEJMoa0806375 PMC2877497

[22] Gyhagen M Bullarbo M Nielsen T. The prevalence of urinary incontinence 20 years after childbirth: a national cohort study in singleton primiparae after vaginal or Caesarean delivery: urinary incontinence 20 years after childbirth. BJOG. 2013;120:144–51. 22413831 10.1111/j.1471-0528.2012.03301.x

[23] Gyhagen M Bullarbo M Nielsen T. A comparison of the long-term consequences of vaginal delivery versus caesarean section on the prevalence, severity and bothersomeness of urinary incontinence subtypes: a national cohort study in primiparous women. BJOG. 2013;120:1548–55. 23786421 10.1111/1471-0528.12367

[24] Ramalingam K Monga A. Obesity and pelvic floor dysfunction. Best Pract Res Clin Obstet Gynaecol. 2015;29:541–7. 25805440 10.1016/j.bpobgyn.2015.02.002

[25] Noblett KL Jensen JK Ostergard DR. The relationship of Body Mass Index to intra-abdominal pressure as measured by multichannel cystometry. Int Urogynecol J Pelvic Floor Dysfunct. 1997;8:323–6. 9609328 10.1007/BF02765589

[26] Marcelissen T Anding R Averbeck M Hanna‐Mitchell A Rahnama'i S Cardozo L. Exploring the relation between obesity and urinary incontinence: pathophysiology, clinical implications, and the effect of weight reduction, ICI‐RS 2018. Neurourol Urodyn. 2019;38(Suppl 5):S18–24. 31821633 10.1002/nau.24072

[27] Brown JS Grady D Ouslander JG, . Prevalence of urinary incontinence and associated risk factors in postmenopausal women. Heart & Estrogen/Progestin Replacement Study (HERS) Research Group. Obstet Gynecol. 1999;94:66–70. 10389720 10.1016/s0029-7844(99)00263-x

[28] Townsend MK Curhan GC Resnick NM Grodstein F. BMI, waist circumference, and incident urinary incontinence in older women. Obesity (Silver Spring, Md.). 2008;16:881–6. 18379564 10.1038/oby.2008.14

